# Synthesis of some novel hydrazono acyclic nucleoside analogues

**DOI:** 10.3762/bjoc.6.49

**Published:** 2010-05-17

**Authors:** Mohammad N Soltani Rad, Ali Khalafi-Nezhad, Somayeh Behrouz

**Affiliations:** 1 Department of Chemistry, Faculty of Basic Sciences, Shiraz University of Technology, Shiraz 71555-313, Iran, Fax: +98-711-7354523; 2 Department of Chemistry, College of Sciences, Shiraz University, Shiraz 71454, Iran

**Keywords:** acyclic nucleoside, chemotherapeutic agent, hydrazone, ketone, miconazole

## Abstract

The syntheses of novel hydrazono acyclic nucleosides similar to miconazole scaffolds are described. In this series of acyclic nucleosides, pyrimidine as well as purine and other azole derivatives replaced the imidazole function in miconazole and the ether group was replaced with a hydrazone moiety using phenylhydrazine. To interpret the dominant formation of (*E*)-hydrazone derivatives rather than (*Z*)-isomers, PM3 semiempirical quantum mechanic calculations were carried out which indicated that the (*E*)-isomers had the lower heats of formation.

## Introduction

In recent years, fungal infections have become an important complication and a major cause of morbidity and mortality in immune-compromised individuals who suffer from tuberculosis, cancer or AIDS, and who undergo organ transplants [1–2]. Up to now, there has been an increase in the number of antifungal drugs available with various structures and scaffolds. However, their clinical value has been limited by the emergence of drug resistance, high risk of toxicity, insufficiencies in their antifungal activity as well as undesirable side effects. This situation has led to an ongoing search for safe and efficient chemotherapeutic agents with potent broad-spectrum antifungal activities.

**Figure 1:**
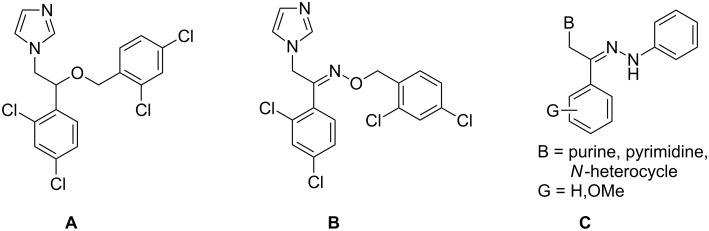
Chemical structures of miconazole (**A**), oxiconazole (**B**) and hydrazono acyclic nucleoside analogue (**C**).

Some of the best known antifungal azole drugs having rational versatility in structures are miconazole (**A**), oxiconazole (**B**) and related compounds, namely aryl azoles (Figure 1).

Pharmacokinetic properties and cellular permeability of a drug can be modulated by derivatization to bio-reversible forms of the drug, namely hydrazones [3]. Hydrazone derivatives are prominent structural motifs in numerous pharmaceutically active compounds. Many well known drugs with various clinical activities, such as chemotherapeutic (e.g. nitrofurantoin [4–5]), antihypertensive (e.g. endralazine [4,6]), antibiotic (e.g. rifampicin [4]), tuberculostatic (e.g. glyconiazide [4,7–8]), skeletal muscle relaxant and antispasmodic (e.g. dantrolene [4,9]), antiseptic (e.g. nitrofural [4]) and intercalating antineoplastic (e.g. bisantrene [4,10]), contain a hydrazone moiety in their structure. Furthermore, various structurally related miconazole bioactive hydrazones are known as antimicrobial and antifungal agents [11–12].

The significance of nucleoside chemistry in drug discovery is well-known and fully established in medicinal chemistry [13–16]. Acyclic nucleosides constitute a special class of nucleoside analogues which have attracted great interest due to their broad-spectrum chemotherapeutic activities against cancer and infections caused by viruses, microbes and other pathogenic microorganisms. Moreover, antitumor and antiviral activities of various hydrazone derivatives of nucleosides have also been reported [16–18].

Inspired by the miconazole scaffold and also as an extension of our ongoing research in the design and synthesis of novel acyclic nucleosides [19–25], hereby, we wish to report the synthesis of some novel hydrazono acyclic nucleosides having similar scaffolds to the miconazole framework. In these compounds, the nucleobases including pyrimidines, purines and other azole derivatives were substituted as heterocyclic cores and the ether bond in miconazole (i.e. CHOCH_2_) was replaced with hydrazono bond (i.e. C=NNH) in the newly synthesized compounds. Figure 1 shows the general structure of hydrazono acyclic nucleoside compound **C**.

## Results and Discussion

**Scheme 1:**
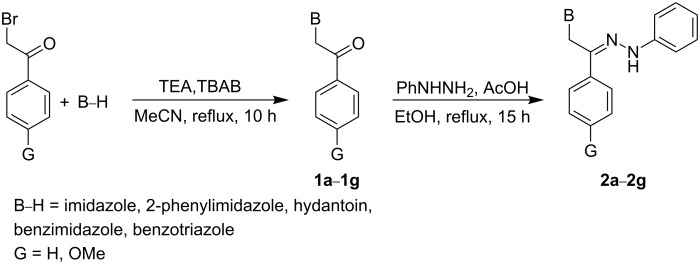
Synthetic pathway for hydrazono acyclic nucleoside **2a**–**2g**.

**Scheme 2:**
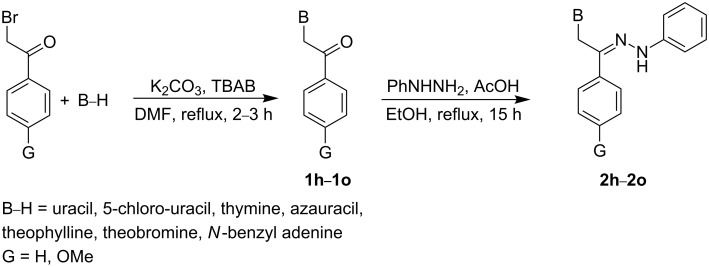
Synthetic pathway for hydrazono acyclic nucleoside **2h**–**2o**.

The synthetic pathways for compounds **2a**–**2g** and **2h**–**2o** are outlined in Scheme 1 and Scheme 2. Two different strategies were considered for the synthesis of the title compounds taking into account the differences in chemical behavior of purine and pyrimidine nucleobases compared to azoles. Because of their better solubility, reactivity and ease of separation of products, the reactions of the azole derivatives were conducted according to Scheme 1.

As shown in Scheme 1, condensation of imidazole, 2-phenylimidazole, hydantoin, benzimidazole and benzotriazole with 2-bromoacetophenone and 4′-methoxy-2-bromoacetophenone gave the key intermediates, ketones **1a**–**1g**. Based on our literature survey, we recognized that among published methods for N-alkylation of imidazole derivatives [26–34], the method developed by Liu et al. [35] was the most appropriate one for N-alkylation of azoles and their derivatives, since in this method the formation of quaternary imidazolium salts is largely prevented. However, from our experience, using triethylamine (TEA) as a homogeneous base instead of K_2_CO_3_ (which was previously employed by Liu et al.) produced more satisfactory results. Hence, the reaction of imidazole or benzimidazole derivatives with 2-bromoacetophenones and TEA in the presence of a catalytic amount of *n*-tetrabutylammonium bromide (TBAB) in refluxing anhydrous acetonitrile (MeCN) provided ketones **1a**–**1g** in good yields (60–70%). Subsequently, the ketones **1a**–**1g** were converted to the pure hydrazone derivatives **2a**–**2g** by treatment with phenylhydrazine in the presence of a catalytic amount of acetic acid in ethanol, followed by heating at reflux (Scheme 1).

For the synthesis of the purine and pyrimidine analogues of target compounds **2h**–**2o**, we envisaged that there might be substantial problems in the synthesis of the desired ketones using similar method as outlined in Scheme 1. This proved to be the case, and the corresponding ketones **1h**–**1o** could not be obtained satisfactorily by this method. The main limitation of using the aforementioned pathway for purines and pyrimidines is their low solubility in acetonitrile, which results in low yields of the corresponding ketones **1h**–**1o**. Thus, we have modified Scheme 1 by using DMF as the solvent of choice for nucleobases (Scheme 2).

Thus, the condensation of purine and pyrimidine nucleobases as well as theophylline and theobromine with 2-bromoacetophenones using K_2_CO_3_ in anhydrous DMF under reflux conditions provides the N-alkylation adducts **1h**–**1o** in moderate yields (Scheme 2). Subsequently, treatment of the obtained ketones **1h**–**1o** with phenylhydrazine in refluxing ethanol in the presence of a catalytic amount of acetic acid afforded the desired compounds **2h**–**2o**. The structures of synthesized compounds are shown in Table 1. As Table 1 indicates, the N-alkylation reactions of nucleobases were achieved regioselectively. In the case of pyrimidine nucleobases, N(1)-alkylated compounds **1h**–**1l** were the major products (43%, 40%, 51%, 47% and 28%); however, N(1),N(3)-dialkylated adduct was also observed in trace amounts (<10%). Moreover, *N*-benzyl adenine derivative **1o** was obtained as the N(9)-isomer in 76% yield, while theophylline was mostly alkylated at N(7) to afford **1m** (69%).

**Table 1:** Synthesized hydrazono acyclic nucleoside analogues.

**Entry**	**Product (2)**	**mp (°C)**	**Yield^a^ (%)**

**a**	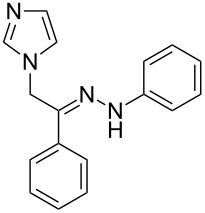	169.2	87
**b**	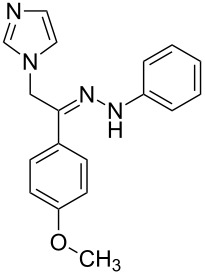	171.3	84
**c**	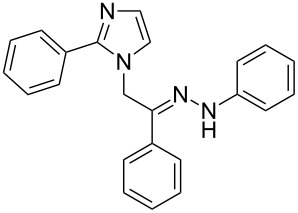	174.5	82
**d**	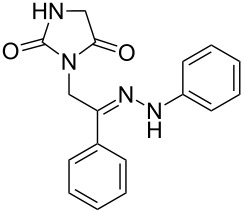	200.1	89
**e**	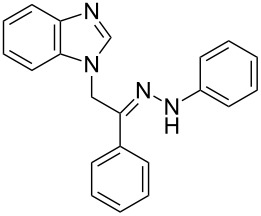	165.9	75
**f**	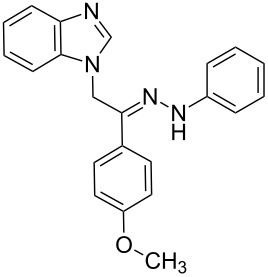	148.4	81
**g**	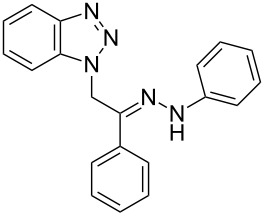	137.9	82
**h**	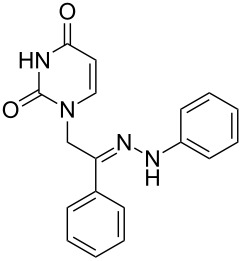	300.1	78
**i**	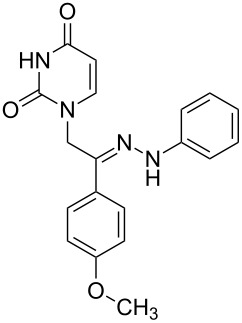	213.8	80
**j**	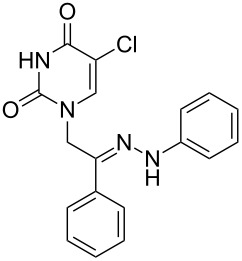	205.1	75
**k**	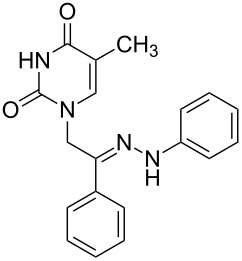	181.5	79
**l**	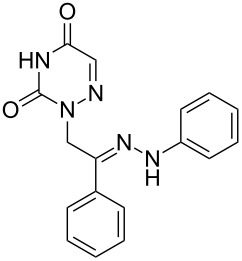	173.9	74
**m**	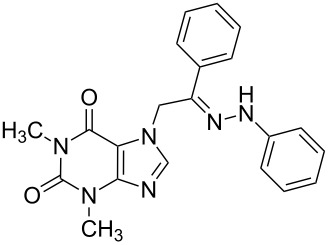	100.1	81
**n**	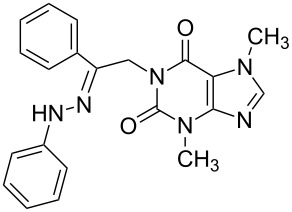	104.7	71
**o**	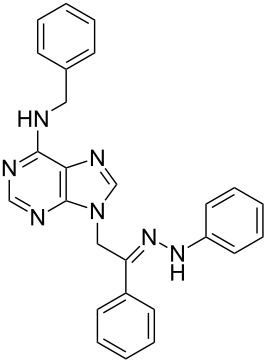	209.3	84

^a^Isolated yield.

All compounds were fully characterized and their structures confirmed by ^1^H and ^13^C NMR, elemental analysis, mass and IR spectroscopy. Although compounds **2a**–**2o** are expected to be produced as two geometrical isomers ((*E*)- or (*Z*)-isomer), ^1^H and ^13^C NMR analysis indicated that the (*E*)-isomer was obtained as major product; the minor (*Z*)-isomer was detected in trace amounts (<5%). To interpret the preference for the (*E*)-isomers, PM3 semiempirical quantum mechanical calculations were carried out using MOPAC in CS Chem 3D Ultra 8 (Cambridge Soft, 2004) or Hyperchem (Hypercube Inc., Version 7). The results are summarized in Table 2; Δ*E* refers to the energy difference between the (*Z*)- and (*E*)-isomer (Δ*E* = *E*_Z_ − *E*_E_ (kcal/mol)). As can be seen in Table 2, calculated Δ*E* values for all hydrazone derivatives **2a**–**2o** are positive. There is accord between the experimental observations and calculated data (Table 2) which supports the greater stability of the (*E*)-isomers over the (*Z*)-isomers and hence predominant formation of the (*E*)-products.

**Table 2:** Calculated heat of formation of synthesized hydrazones **2a**–**2o** using PM3.

**Entry**	***E*_E_^a^**	***E*_Z_^b^**	**Δ*E*^c^**

**2a**	123.87244	129.56016	5.68772
**2b**	85.14540	91.62108	6.47568
**2c**	148.47301	153.04860	4.57559
**2d**	14.42535	21.51980	7.09445
**2e**	142.60529	146.16960	3.56431
**2f**	103.74787	117.44982	13.70195
**2g**	183.57186	198.25266	14.68080
**2h**	26.07071	28.66346	2.59275
**2i**	−13.34542	−9.56043	3.78499
**2j**	21.58339	24.58239	2.99900
**2k**	17.75077	20.31085	2.56008
**2l**	52.80499	55.83149	3.02560
**2m**	21.96581	45.90909	23.94328
**2n**	52.57276	60.37796	7.80520
**2o**	176.15539	185.18056	9.02517

^a^Heat of formation of (*E*)-isomer (kcal/mol). ^b^Heat of formation of (*Z*)-isomer (kcal/mol). ^c^Δ*E* = *E*_Z_ − *E*_E_ (kcal/mol).

## Conclusion

In summary, we have synthesized novel acyclic nucleosides **2a**–**2o** containing a miconazole-like scaffold as well as hydrazone moiety. In this series of acyclic nucleosides, we employed various nucleobases and other imidazole derivatives in place of the imidazole in miconazole. To interpret the dominant formation of the (*E*)-hydrazone derivatives rather than the (*Z*)-isomers, PM3 semiempirical quantum mechanical calculations were carried out which showed that the heats of formation of the (*E*)-isomers were lower.

The biological studies of compounds **2a**–**2o** are currently under investigation and will be reported in due course.

## Experimental

### General

All chemicals were purchased from Fluka or Merck. Solvents were purified and dried according to the reported methods [36] and stored over 0.3 nm molecular sieves. The progress of reaction was followed by TLC using silica gel SILG/UV 254 plates. Silica gel 60, 0.063–0.200 mm (70–230 mesh ASTM) was used for column chromatography. IR spectra were run on a Shimadzu FTIR-8300 spectrophotometer. The ^1^H NMR (250 MHz) and ^13^C NMR (62.5 MHz) were run on a Bruker Avance DPX-250, FT-NMR spectrometer (δ in ppm, *J* in Hz). Mass spectra were recorded on a Shimadzu GC MS-QP 1000 EX apparatus. Microanalyses were performed on a Perkin-Elmer 240-B microanalyzer. Melting points (mp) were recorded on a Büchi 510 apparatus in open capillary tubes and are uncorrected.

#### General procedure for the synthesis of ketones **1a–1g**

In a two-neck round-bottom flask (100 mL) equipped with a condenser, a mixture of the appropriate *N*-heterocycle (0.01 mol), the 2-bromoacetophenone (0.012 mol), anhydrous TEA (1.01 g, 0.01 mol) and a catalytic amount of TBAB (0.1 g) was dissolved in dry acetonitrile (40 mL). The mixture was then heated at reflux for 10 h (TLC control). The solvent was evaporated at reduced pressure, the residue dissolved in CHCl_3_ (200 mL) and washed with H_2_O (2 × 100 mL). The organic layer was dried (10 g of Na_2_SO_4_) and concentrated to afford the crude product, which was purified by column chromatography on silica gel eluting with an appropriate solvent.

#### General procedure for the synthesis of ketones **1h–1o**

In a two-neck round-bottom flask (100 mL) equipped with a condenser, a mixture of the appropriate nucleobase (0.01 mol), the 2-bromoacetophenone (0.012 mol), K_2_CO_3_ (1.38 g, 0.01 mol) and a catalytic amount of TBAB (0.1 g) was dissolved in dry DMF (30 mL). The mixture was then heated at reflux for 2–3 h (TLC control). The solvent was evaporated at reduced pressure, the residue dissolved in CHCl_3_ (200 mL) and washed with H_2_O (2 × 100 mL). The organic layer was dried (10 g of Na_2_SO_4_) and concentrated to afford the crude product, which was purified by column chromatography on silica gel eluting with an appropriate solvent.

#### General procedure for the synthesis of hydrazono acyclic nucleoside analogues **2a–2o**

In a two-neck round-bottom flask (100 mL) equipped with a condenser, a mixture of the appropriate ketone **1a**–**1o** (0.01 mol), phenylhydrazine (1.62 g, 0.015 mol) and acetic acid (3 drops) was dissolved in ethanol (15 mL) and the mixture heated at reflux for 15 h (TLC control). The reaction mixture was then stored in a refrigerator overnight. It was filtered and washed with cold ethanol (2 × 5 mL) and recrystallized from MeOH/H_2_O to afford the pure phenylhydrazone derivatives.

## Supporting Information

Supporting information features physical and spectroscopic data for all novel compounds **2a**–**2o**.

File 1 Synthesis of some novel hydrazono acyclic nucleoside analogues
