# Supplementary material for: Synthesis of some novel hydrazono acyclic nucleoside analogues
Source: Beilstein J Org Chem. 2010 May 17;6:49. doi: 10.3762/bjoc.6.49 (PMC2887276; doi:10.3762/bjoc.6.49)
Supplement: File 1 — Synthesis of some novel hydrazono acyclic nucleoside analogues [file Beilstein_J_Org_Chem-06-49-s001.pdf]

Supporting Information

for

Synthesis of some novel hydrazono acyclic nucleoside analogues

Mohammad N. Soltani Rad*[1], Ali Khalfi-Nezhad[2] and Somayeh Behrouz[1]

Address: [1]Department of Chemistry, Faculty of Basic Sciences, Shiraz University of Technology, Shiraz 71555-313, Iran, Fax: +98-711-7354523 and [2]Department of Chemistry, College of Sciences, Shiraz University, Shiraz 71454, Iran

Email: Mohammad N. Soltani Rad - soltani@sutech.ac.ir; nsoltanirad@gmail.com

* Corresponding author

1-[2-(1*H*-Imidazole-1-yl)-1-phenylethylidene]-2-phenylhydrazine (2a)

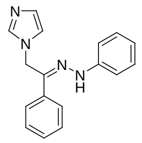

Recrystallization from MeOH/H_2O afforded the pure product as yellow crystals (2.4 g, 87%); mp = 169.2 °C; R_f = 0.37 (EtOAc).

^{1}H NMR (250 MHz, DMSO-d_6): δ = 10.25 (s, 1H, N*H*, exchangeable with D_2O), 7.82–6.82 (complex, 13H, aryl, imidazole), 5.45 (s, 2H, CH_2).

^{13}C NMR (62.5 MHz, DMSO-d_6): δ = 39.48, 113.07, 119.18, 119.81, 125.20, 127.73, 128.40, 128.52, 129.06, 136.51, 137.01, 137.15, 145.32.

Anal. Calcd for $C_{17}H_{16}N_4$: C, 73.89; H, 5.84; N, 20.27. Found: C, 73.91; H, 5.87; N, 20.22.

IR (KBr) ν (cm^{-1}): 3201.6, 3093.6, 2962.5, 1596.9, 1488.9.

MS (EI) [*m*/*z* (%)]: 276.1 (22.4) [M^+].

1-[2-(1*H*-Imidazole-1-yl)-1-(4-methoxy phenyl)ethylidene]-2-phenylhydrazine (2b)

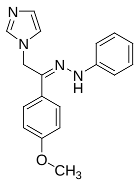

Recrystallization from MeOH/H_2O afforded the pure product as yellow crystals (2.57 g, 84%); mp = 171.3 °C; R_f = 0.35 (EtOAc).

^{1}H NMR (250 MHz, DMSO-d_6): δ = 10.09 (s, 1H, N*H*, exchangeable with D_2O), 7.73–6.83 (complex, 12H, aryl, imidazole), 5.43 (s, 2H, CH_2), 3.76 (s, 3H, OCH_3).

^{13}C NMR (62.5 MHz, DMSO-d_6): δ = 44.70, 60.30, 118.11, 119.07, 124.41, 124.68, 131.87, 133.71, 134.25, 134.78, 142.01, 142.37, 150.77, 164.35.

Anal. Calcd for $C_{18}H_{18}N_4O$: C, 70.57; H, 5.92; N, 18.29. Found: C, 70.61; H, 5.88; N, 18.32.

IR (KBr) ν (cm^{-1}): 3224.8, 3109.0, 2916.2, 1604.1, 1573.8.

MS (EI) [*m*/*z* (%)]: 306.1 (24.6) [M^+].

1-Phenyl-2-[1-phenyl-2-(2-phenyl-1*H*-imidazole-1-yl)ethylidene]hydrazine (2c)

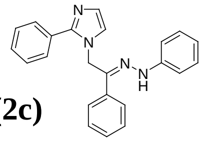

Recrystallization from MeOH/H_2O afforded the pure product as yellow crystals (2.88 g, 82%); mp = 174.5 °C; R_f = 0.66 (EtOAc).

^{1}H NMR (250 MHz, DMSO-d_6): δ = 10.24 (s, 1H, N*H*, exchangeable with D_2O), 7.73–6.85 (complex, 17H, aryl, imidazole), 5.46 (s, 2H, CH_2).

^{13}C NMR (62.5 MHz, DMSO-d_6): δ = 41.49, 113.06, 113.55, 119.79, 120.75, 125.06, 127.59, 128.11, 128.17, 128.40, 128.48, 129.04, 130.58, 136.62, 136.91, 145.35, 146.58.

Anal. Calcd for $C_{23}H_{20}N_4$: C, 78.38; H, 5.72; N, 15.90. Found: C, 78.42; H, 5.67; N, 15.93.

IR (KBr) ν (cm^{-1}): 3224.8, 3047.3, 2939.3, 1591.9, 1465.8.

MS (EI) [*m*/*z* (%)]: 352.2 (31.3) [M^+].

3-[2-Phenyl-2-(phenylhydrazono)ethyl]-imidazolidine-2,4-dione (2d) 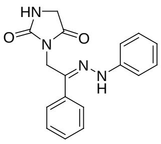

Recrystallization from MeOH/H_2O afforded the pure product as pale-yellow crystals (2.74 g, 89%); mp = 200.1 °C; R_f = 0.77 (EtOAc).

^{1}H NMR (250 MHz, DMSO-d_6): δ = 10.07 (s, 1H, N*H*, exchangeable with D_2O), 8.37 (s, 1H, N1-*H*, hydantoin, exchangeable with D_2O), 7.91–6.80 (complex, 10H, aryl), 4.68 (s, 2H, CH_2), 3.94 (s, 2H, N1-CH_2).

^{13}C NMR (62.5 MHz, DMSO-d_6): δ = 33.04, 46.04, 112.77, 119.65, 125.74, 127.62, 128.12, 129.06, 137.18, 137.69, 145.36, 157.76, 172.31.

Anal. Calcd for $C_{17}H_{16}N_4O_2$: C, 66.22; H, 5.23; N, 18.17. Found: C, 66.25; H, 5.26; N, 18.21.

IR (KBr) ν (cm^{-1}): 3386.8, 3170.8, 3042.5, 2831.3, 1712.7, 1593.1, 1465.8.

MS (EI) [*m/z* (%)]: 308.1 (29.8) [M^+].

1-[2-(1*H*-Benzo[*d*]imidazol-1-yl)-1-phenylethylidene]-2-phenylhydrazine (2e) 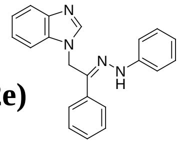

Recrystallization from MeOH/H_2O afforded the pure product as pale-yellow crystals (2.44 g, 75%); mp = 165.9 °C; R_f = 0.59 (EtOAc).

^{1}H NMR (250 MHz, DMSO-d_6): δ = 10.41 (s, 1H, N*H*, exchangeable with D_2O), 8.26 (s, 1H, C(2)-H, benzimidazole), 7.80–6.88 (complex, 14H, aryl), 5.74 (s, 2H, CH_2).

^{13}C NMR (62.5 MHz, DMSO-d_6): δ = 39.77, 108.89, 111.47, 114.41, 120.56, 123.94, 124.22, 126.71, 126.99, 127.11, 127.95, 129.05, 129.63, 130.44, 136.36, 142.16, 145.43.

Anal. Calcd for $C_{21}H_{18}N_4$: C, 77.28; H, 5.56; N, 17.17. Found: C, 77.32; H, 5.59; N, 17.12.

IR (KBr) ν (cm^{-1}): 3158.2, 3037.7, 2983.7, 1583.4, 1492.8.

MS (EI) [*m/z* (%)]: 326.1 (19.8) [M^+].

1-[2-(1*H*-Benzo[*d*]imidazol-1-yl)-1-(4-methoxyphenyl)ethylidene]-2-phenylhydrazine (2f)

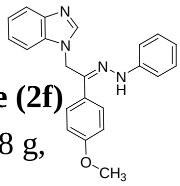

Recrystallization from MeOH/H_2O afforded the pure product as pale-yellow crystals (2.88 g, 81%); mp = 148.4 °C; R_f = 0.47 (EtOAc).

^{1}H NMR (250 MHz, DMSO-d_6): δ = 9.06 (s, 1H, N*H*, exchangeable with D_2O), 8.09 (s, 1H, C(2)-H, benzimidazole), 7.62–6.71 (complex, 13H, aryl), 5.37 (s, 2H, CH_2), 3.75 (s, 3H, OCH_3).

^{13}C NMR (62.5 MHz, DMSO-d_6): δ = 50.71, 55.03, 110.70, 112.73, 114.10, 114.41, 119.30, 121.42, 122.30, 123.85, 128.68, 129.26, 134.03, 139.57, 143.35, 144.32, 145.54, 159.42.

Anal. Calcd for $C_{22}H_{20}N_4O$: C, 74.14; H, 5.66; N, 15.72. Found: C, 74.19; H, 5.72; N, 15.75.

IR (KBr) ν (cm^{-1}): 3207.4, 3041.5, 2897.6, 1579.4, 1456.8.

MS (EI) [*m*/*z* (%)]: 356.2 (34.0) [M^+].

1-[2-(1*H*-Benzo[*d*][1,2,3]triazole-1-yl)-1-phenylethylidene]-2-phenylhydrazine (2g)

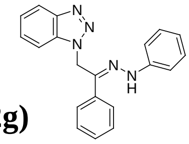

Recrystallization from MeOH/H_2O afforded the pure product as scarlet crystals (2.68 g, 82%); mp = 137.9 °C; R_f = 0.90 (EtOAc).

^{1}H NMR (250 MHz, DMSO-d_6): δ = 9.20 (s, 1H, N*H*, exchangeable with D_2O), 8.03–6.71 (complex, 14H, aryl), 5.88 (s, 2H, CH_2).

^{13}C NMR (62.5 MHz, DMSO-d_6): δ = 53.90, 110.75, 112.76, 119.04, 119.23, 123.77, 127.19, 127.82, 128.67, 128.98, 129.04, 131.84, 133.19, 138.45, 145.15, 145.29.

Anal. Calcd for $C_{20}H_{17}N_5$: C, 73.37; H, 5.23; N, 21.39. Found: C, 73.42; H, 5.27; N, 21.41.

IR (KBr) ν (cm^{-1}): 3301.9, 3051.2, 2914.2, 1598.9, 1490.4.

MS (EI) [*m*/*z* (%)]: 327.1 (15.7) [M^+].

1-[2-Phenyl-2-(phenylhydrazono)ethyl]-1*H*-pyrimidine-2,4-dione (2h) 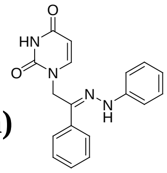

Recrystallization from MeOH/H_2O afforded the pure product as bright brown crystals (2.49 g, 78%); mp = 300.1 °C; R_f = 0.52 (EtOAc).

^1H NMR (250 MHz, DMSO-d_6): δ = 10.99 (s, 1H, N3-*H*, uracil, exchangeable with D_2O), 8.80 (s, 1H, NH, exchangeable with D_2O), 7.42 (d, 1H, *J* = 6.7 Hz, C(6)-*H*, uracil), 7.23–6.45 (complex, 10H, aryl), 5.36 (d, 1H, *J* = 6.7 Hz, C(5)-*H*, uracil), 4.49 (s, 2H, CH_2).

^{13}C NMR (62.5 MHz, DMSO-d_6): δ = 51.57, 100.41, 112.50, 118.91, 127.69, 128.62, 129.14, 132.35, 139.20, 140.16, 145.59, 146.49, 150.96, 163.88.

Anal. Calcd for $C_{18}H_{16}N_4O_2$: C, 67.49; H, 5.03; N, 17.49. Found: C, 67.44; H, 4.99; N, 17.43.

IR (KBr) ν (cm^{-1}): 3294.2, 3162.5, 3042.5, 2831.3, 1720.2, 1712.7, 1596.9.

MS (EI) [*m*/*z* (%)]: 320.1 (35.2) [M^+].

1-[2-(4-Methoxyphenyl)-2-(phenylhydrazono)ethyl]-1*H*-pyrimidine-2,4-dione (2i) 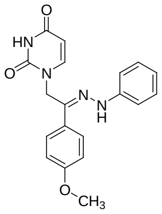

Recrystallization from MeOH/H_2O afforded the pure product as bright brown crystals (2.80 g, 80%); mp = 213.8 °C; R_f = 0.50 (EtOAc) 0.50.

^1H NMR (250 MHz, DMSO-d_6): δ = 11.27 (s, 1H, N3-*H*, uracil, exchangeable with D_2O), 8.97 (s, 1H, N*H*, exchangeable with D_2O), 7.61 (d, 1H, *J* = 6.5 Hz, C(6)-*H*, uracil), 7.42–6.68 (complex, 9H, aryl), 5.59 (d, 1H, *J* = 6.5 Hz, C(5)-*H*, uracil), 4.70 (s, 2H, CH_2), 3.79 (s, 3H, OCH_3).

^{13}C NMR (62.5 MHz, DMSO-d_6): δ = 51.57, 55.07, 100.41, 112.48, 114.54, 118.80, 124.30, 128.63, 129.00, 139.22, 145.71, 146.46, 151.27, 159.60, 163.89.

Anal. Calcd for $C_{19}H_{18}N_4O_3$: C, 65.13; H, 5.18; N, 15.99. Found: C, 65.15; H, 5.21; N, 16.02.

IR (KBr) ν (cm^{-1}): 3261.4, 3170.8, 3028.0, 2912.3, 1730.1, 1715.6, 1558.4.

MS (EI) [*m*/*z* (%)]: 350.1 (33.4) [M^+].

5-Chloro-1-[2-phenyl-2-(phenylhydrazono)ethyl]-1*H*-pyrimidine-2,4-dione (2j) 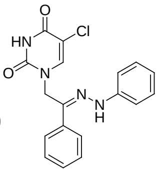

Recrystallization from MeOH/H_2O afforded the pure product as pale-yellow crystals (2.69 g, 76%); mp = 205.1 °C; R_f = 0.83 (EtOAc).

^{1}H NMR (250 MHz, DMSO-d_6): δ = 11.87 (s, 1H, N3-*H*, uracil, exchangeable with D_2O), 9.07 (s, 1H, NH, exchangeable with D_2O), 8.19 (s, 1H, C(6)-*H*, uracil), 7.56–6.65 (complex, 10H, aryl), 4.72 (s, 2H, CH_2).

^{13}C NMR (62.5 MHz, DMSO-d_6): δ = 56.79, 105.64, 117.73, 124.14, 132.93, 133.86, 134.39, 137.60, 144.44, 145.71, 150.84, 151.73, 156.19, 169.13.

Anal. Calcd for $C_{18}H_{15}ClN_4O_2$: C, 60.94; H, 4.26; Cl, 9.99; N, 15.79. Found: C, 61.01; H, 4.30; Cl, 9.93; N, 15.82.

IR (KBr) ν (cm^{-1}): 3481.3, 3284.5, 3060.8, 2960.5, 1725.1, 1715.9, 1596.7, 758.0.

MS (EI) [*m*/*z* (%)]: 354.1 (29.9) [M^+].

5-Methyl-1-[2-phenyl-2-(phenylhydrazono)ethyl]-1*H*-pyrimidine-2,4-dione (2k) 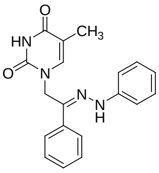

Recrystallization from MeOH/H_2O afforded the pure product as yellow crystals (2.64 g, 79%); mp = 181.5 °C; R_f = 0.70 (EtOAc).

^{1}H NMR (250 MHz, DMSO-d_6): δ = 11.15 (s, 1H, *N*3-*H*, thymine, exchangeable with D_2O), 8.90 (s, 1H, N*H*, exchangeable with D_2O), 7.47–6.85 (complex, 10H, aryl), 6.61 (s, 1H, C(6)-*H*, thymine), 4.60 (s, 2H, CH_2), 3.38 (s, 3H, CH_3).

^{13}C NMR (62.5 MHz, DMSO-d_6): δ = 24.39, 51.35, 107.89, 112.51, 118.91, 127.66, 128.62, 129.13, 132.31, 139.36, 142.26, 145.61, 151.01, 164.49, 177.74.

Anal. Calcd for $C_{19}H_{18}N_4O_2$: C, 68.25; H, 5.43; N, 16.76. Found: C, 68.21; H, 5.42; N, 16.79.

IR (KBr) ν (cm^{-1}): 3286.5, 3178.5, 3034.6, 2908.4, 1720.4, 1708.1, 1592.8.

MS (EI) [*m*/*z* (%)]: 334.1 (36.5) [M^+].

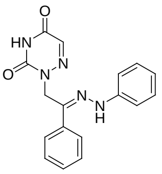

2-[2-Phenyl-2-(phenylhydrazono)ethyl]-2*H*-[1,2,4]triazine-3,5-dione (2l)

Recrystallization from MeOH/H_2O afforded the pure product as pale-yellow crystals (2.37 g, 74%); mp = 173.9 °C; R_f = 0.80 (EtOAc).

^{1}H NMR (250 MHz, DMSO-d_6): δ = 12.65 (s, 1H, *N*3-*H*, azauracil, exchangeable with D_2O), 9.74 (s, 1H, N*H*, exchangeable with D_2O), 7.50–7.08 (complex, 10H, aryl), 6.79 (s, 1H, C(5)-*H*, azauracil), 5.07 (s, 2H, CH_2).

^{13}C NMR (62.5 MHz, DMSO-d_6): δ = 35.26, 112.77, 119.40, 126.03, 127.67, 127.97, 129.16, 134.53, 136.77, 138.53, 145.73, 149.09, 159.17.

Anal. Calcd for $C_{17}H_{15}N_5O_2$: C, 63.54; H, 4.71; N, 21.79. Found: C, 63.60; H, 4.66; N, 21.82.

IR (KBr) ν (cm^{-1}): 3244.0, 3072.4, 3033.8, 2954.5, 1728.1, 1710.5, 1583.8.

MS (EI) [*m*/*z* (%)]: 321.1 (18.2) [M^+].

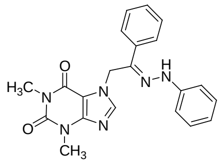

1,3-Dimethyl-7-[2-phenyl-2-(phenylhydrazono)ethyl]-3,7-dihydro-purine-2,6-dione (2m)

Recrystallization from MeOH/H_2O afforded the pure product as pale-yellow crystals (3.14 g, 81%); mp = 100.1 °C; R_f = 0.74 (EtOAc).

^{1}H NMR (250 MHz, DMSO-d_6): δ = 10.25 (s, 1H, C(8)-*H*, theophylline), 7.78 (s, 1H, N*H*, exchangeable with D_2O), 7.70–6.80 (complex, 10H, aryl), 5.67 (s, 2H, CH_2), 3.37 (s, 6H, *N*1-Me and N3-Me).

^{13}C NMR (62.5 MHz, DMSO-d_6): δ = 27.60, 29.34, 50.66, 106.20, 112.99, 119.79, 125.43, 127.77, 128.38, 128.98, 132.13, 135.82, 141.78, 145.22, 148.09, 150.76, 154.95.

Anal. Calcd for $C_{21}H_{20}N_6O_2$: C, 64.94; H, 5.19; N, 21.64. Found: C, 64.91; H, 5.22; N, 21.61.

IR (KBr) ν (cm^{-1}): 3340.5, 3025.1, 2954.7, 1720.7, 1708.9, 1591.4.

MS (EI) [*m*/*z* (%)]: 388.2 (41.1) [M^+].

3,7-Dimethyl-1-[2-phenyl-2-(phenylhydrazono)ethyl]-3,7-dihydro-purine-2,6-dione (2n)

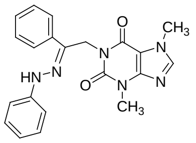

Recrystallization from MeOH/H_2O afforded the pure product as pale-yellow crystals (2.75 g, 71%); mp = 104.7 °C; R_f = 0.44 (EtOAc).

^{1}H NMR (250 MHz, DMSO-d_6): δ = 9.58 (s, 1H, C(8)-*H*, theobromine), 8.64 (s, 1H, N*H*, exchangeable with D_2O), 7.83–6.41 (complex, 10H, aryl), 4.99 (s, 2H, CH_2), 3.64 (s, 3H, *N*3-Me), 3.21 (s, 3H, *N*7-Me).

^{13}C NMR (62.5 MHz, DMSO-d_6): δ = 29.31, 33.10, 44.88, 106.49, 112.60, 118.55, 126.02, 127.86, 129.07, 132.92, 137.30, 139.82, 143.37, 145.65, 148.30, 150.92, 154.41.

Anal. Calcd for $C_{21}H_{20}N_6O_2$: C, 64.94; H, 5.19; N, 21.64. Found: C, 64.91; H, 5.20; N, 21.62.

IR (KBr) ν (cm^{-1}): 3253.7, 3045.4, 2945.1, 1725.2, 1705.0, 1593.8.

MS (EI) [*m*/*z* (%)]: 388.2 (43.7) [M^+].

Benzyl-{9-[2-phenyl-2-(phenylhydrazono)ethyl]-9*H*-purine-6-yl}amine (2o)

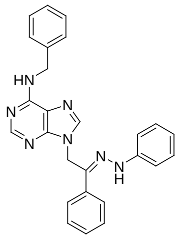

Recrystallization from MeOH/H_2O afforded the pure product as pale-yellow crystals (3.65 g, 84%); mp = 209.3 °C; R_f = 0.75 (EtOAc).

^{1}H NMR (250 MHz, DMSO-d_6): δ = 10.67 (s, 1H, N*H*, exchangeable with D_2O), 8.47–7.12 (complex, 17H, aryl, C(2)-H and C(8)-H, purine), 6.87 (s, 1H, N*H*, purine, exchangeable with D_2O), 5.54 (s, 2H, CH_2, benzyl), 4.71 (s, 2H, CH_2).

^{13}C NMR (62.5 MHz, DMSO-d_6): δ = 37.00, 42.91, 113.08, 118.54, 119.90, 125.34, 126.55, 127.09, 127.85, 128.12, 128.44, 129.12, 136.62, 137.11, 139.88, 140.04, 141.09, 145.44, 152.42, 154.38.

Anal. Calcd for $C_{26}H_{23}N_7$: C, 72.04; H, 5.35; N, 22.62. Found: C, 72.01; H, 5.31; N, 22.59.

IR (KBr) ν (cm^{-1}): 3263.3, 3193.9, 3018.4, 2918.1, 1592.6.

MS (EI) [*m*/*z* (%)]: 433.2 (38.4) [M^+].
